# Actomyosin ring driven cytokinesis in budding yeast

**DOI:** 10.1016/j.semcdb.2016.01.043

**Published:** 2016-05

**Authors:** Franz Meitinger, Saravanan Palani

**Affiliations:** a Ludwig Institute for Cancer Research, Department of Cellular and Molecular Medicine, University of California San Diego, 9500 Gilman Drive, CMM East, La Jolla, CA 92093, United States; b Division of Biomedical Cell Biology, Warwick Medical School, University of Warwick, Coventry CV4 7AL, United Kingdom

**Keywords:** AMR, actomyosin-ring, MEN, mitotic exit network, GEF, guanine nucleotide exchange factor, APC, anaphase promoting complex, Cytokinesis, Actomyosin-ring, Budding yeast, Cell division

## Abstract

Cytokinesis is the final process in the cell cycle that physically divides one cell into two. In budding yeast, cytokinesis is driven by a contractile actomyosin ring (AMR) and the simultaneous formation of a primary septum, which serves as template for cell wall deposition. AMR assembly, constriction, primary septum formation and cell wall deposition are successive processes and tightly coupled to cell cycle progression to ensure the correct distribution of genetic material and cell organelles among the two rising cells prior to cell division. The role of the AMR in cytokinesis and the molecular mechanisms that drive AMR constriction and septation are the focus of current research. This review summarizes the recent progresses in our understanding of how budding yeast cells orchestrate the multitude of molecular mechanisms that control AMR driven cytokinesis in a spatio-temporal manner to achieve an error free cell division.

## 1 Introduction

Cell division is the process that divides one cell into two cells each containing an equal amount of organellar and genetic material. The physical separation of the two daughter cells is called cytokinesis. Cytokinesis involves a periodic interplay between biochemical processes and cellular mechanics [1] and is tightly coupled to cell cycle progression and mitosis to ensure duplication, and correct distribution of genetic material and cell organelles amongst the two daughter cells before they are physically separated [2], [3]. Defects in cell separation or in the temporal coordination of cytokinesis with key cell cycle events can lead to genomic instability/aneuploidy, which can result in reduced fitness, cancer or cell death [4]. Thus, cells have developed robust systems that ensure high fidelity in coordinating cell division processes. In animal and yeast cells, cytokinesis is driven by a contractile actomyosin ring (AMR) [2], [5], [6]. Most components of the AMR and associated proteins are highly conserved across species [7], [8], however, the function of the AMR is optimized to meet the special requirements of the particular organism/cell type. In comparing animal and yeast cells, there are at least two major differences that require specialization of the cell division machinery, cell size and cell cortex. Animal cells usually have a cell diameter of 10–30 μm, whereas budding yeast cells are just 1 μm thick at the cell division site. Consequently, animal cells have to form a larger AMR than yeast cells. Despite the size differences, cultured human cells and budding yeast cells close their cell division site with similar timing (approximately 5 min). Thus, AMR constriction in both systems might work differently. Animal and yeast cells are also different in respect to the intracellular actomyosin cortex present in animal cells and the extracellular cell wall material in yeast cells [9]. Both systems have shaping and stabilization functions. During cell division, animal cells must reorganize their actomyosin cortex to form a contractile AMR exclusively at the cell equator. Yeast cells lack an actomyosin cortex, but instead couple AMR constriction to cell wall/septum formation to maintain cell integrity after cleavage. These major differences may help to explain why the motor domain of the myosin-II heavy chain, which is the basis for contractility and force generation, is essential in animal cells, but non-essential in budding yeast cells [10], [11], [12]. Indeed, it appears that the AMR in budding yeast mainly operates as a scaffold that shrinks in a controlled manner to drive plasma membrane ingression and septum formation [13]. In this model, the function of the contractile AMR is to promote and guide primary septum formation rather than providing the force that pulls the plasma membrane together. This review covers the recent progress of our understanding of how budding yeast cells perform cytokinesis with a focus on the role of the AMR.

## 2 AMR assembly

The core of the AMR is comprised of actin filaments, myosin-II heavy chain (Myo1), essential and regulatory myosin light chains (Mlc1 and Mlc2) and associated proteins, which coordinate AMR constriction with septum formation (see Table 1 for a list of the major proteins involved in cytokinesis). Assembly of the core AMR depends on septins, the Rho-GTPase Rho1, formins Bnr1 and Bni1, the IQGAP-homolog Iqg1, tropomyosins (Tpm1 and Tpm2), profilin and a number of cell cycle regulated kinases and phosphatases (see Table 2 for a list of kinases and phosphatases that regulate cytokinesis).

**Fig. 1:**
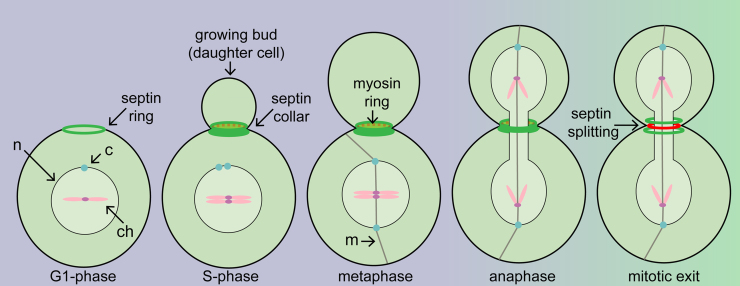
Illustration of cytokinetic key events in different cell cycle phases in budding yeast cells. The septin ring assembles at the cell periphery during G1-phase. A dynamic myosin ring (red dotted ring) forms during G1-S-phase in a septin dependent manner. The septin hour-glass like ring splits into two rings during mitotic exit and sandwiches a stable actomyosin-ring (red ring). c, centrosome; ch, chromosomes; m, microtubules; n, nucleus.

AMR assembly occurs stepwise throughout the cell cycle and is linked to cell cycle signals [3]. The site of AMR assembly is determined at G1-phase when a new bud forms at the cell periphery of the mother cell (Fig. 1). The bud neck between mother and bud (daughter) denotes the future site of cell division (equivalent to mammalian cleavage furrow) and harbors many protein complexes throughout the cell cycle [14]. Some of the first components that assemble at the bud neck, and which are crucial for the stepwise assembly of the AMR, are septins (Fig. 1). Septins are GTP-binding proteins that form filaments that are organized in higher order structures (reviewed in Refs. [14], [15], [16], [17], [18]), and whose assembly at the division site is dependent on the Rho-GTPase Cdc42 [19]. In budding yeast, five septins (Cdc3, Cdc10, Cdc11, Cdc12 and Shs1) are involved in forming hetero-octameric rods [20], [21]. The ability of septins to form filaments is essential [22] and promoted by lipid interaction [23]. Septin filaments form initially at the bud neck into an hourglass like structure, which splits upon mitotic exit into two separate rings that sandwich the AMR (Fig. 1) [24], [25]. Septin splitting is tightly linked to cell cycle progression and a prerequisite for AMR contraction [24]. Recent work has given insight into the molecular reorganization of septin filaments during the splitting of the hourglass like structure into two separated rings upon mitotic exit [21], [26], [27], [28], [29], [30].

**Fig. 2:**
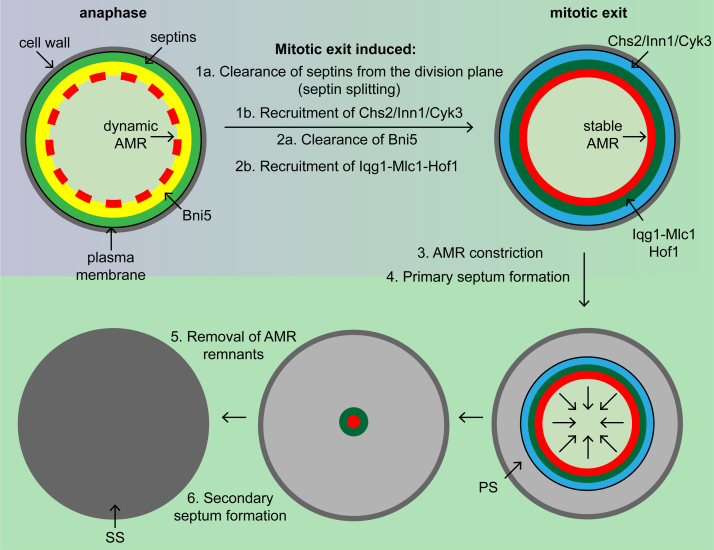
Illustration of the molecular reorganization at the cell division site during mitotic exit (see text for details). AMR, actomyosin-ring; PS, primary septum; SS, secondary septum.

Septins serve as a scaffold for AMR assembly and localization of other proteins, which are involved in cell cycle regulation and cell polarity [14], [18]. Bni5 is one of the earliest proteins recruited by the septin scaffold [30], [31], [32]. Bni5 contributes to the formation of a septin network by crosslinking septin filaments [30]. Efficient recruitment of Bni5 depends on the C-terminal extensions of the septin’s Cdc11 and Shs1 [32]. Importantly, Bni5 is responsible for recruiting Myo1 to the cell division site before mitotic exit [12]. At this step Bni5 and the septin scaffold link the myosin ring with the plasma membrane, however, the molecules involved in this linkage are not fully known (Fig. 2) [12]. Fang et al. have demonstrated that a specific region in the tail of Myo1 (TD1) binds to Bni5 and this interaction is important for the recruitment of Myo1 to the cell division site before the onset of cytokinesis. However, Myo1 localization during cytokinesis is independent of Bni5, which disappears from the cell division site upon mitotic exit (Fig. 2), and depends on a second targeting domain in the tail of Myo1 (TD2) and the protein Iqg1 (the yeast homolog of human IQGAP) [12]. Whether Iqg1 interacts directly with Myo1-TD2 is not known. However, it is likely that Iqg1 contributes at this time to anchor the myosin ring to the plasma membrane (Fig. 2). How Myo1 switches the connection at the cell division site from Bni5 to Iqg1 remains elusive. Fluorescence recovery after photo-bleaching (FRAP) experiments show that Myo1 is mobile at the cell division site and becomes immobilized just before the onset of cytokinesis (Fig. 2) [13]. The stabilization of Myo1 is independent of the motor domain, but dependent on a small region in the tail, which is thought to allow the formation of higher-order/supra molecular complex at the division site [12], [13]. Wloka et al. suggest that this structure acts as scaffold to stabilize proteins involved in septum formation during cytokinesis, such as Inn1, Iqg1, Mlc1 and the F-BAR protein Hof1.

Iqg1 is the sole and essential yeast homolog of IQGAP [33] and is targeted to the cell division site by a cluster of IQ motifs [34]. This cluster interacts directly with Mlc1 [35], [36], which in turn is essential for the recruitment of Iqg1 to the cell division site in late anaphase [37]. An N-terminal calponin-homolog (CH) domain interacts with actin filaments and is crucial for actin ring assembly [34], [38], [39]. In addition, Iqg1 contains a poorly understood RasGAP-related and RasGap-C-terminus-related domain, both of which are implicated in targeting Myo1 to the cell division site [12]. Iqg1 assembly at the cell division site is regulated by Cdk1 dependent phosphorylation [40]. The phosphatase Cdc14, which is activated by the mitotic exit network during telophase [3], counteracts Cdk1 and dephosphorylates Iqg1, thereby contributing to AMR constriction [41]. Iqg1 is also regulated by ubiquitination through the E3 ubiquitin ligase APC (anaphase promoting complex), which targets the protein for proteasome-mediated degradation [42].

As mentioned earlier, Iqg1 is targeted to the cell division site by Mlc1, though it is not clear how Mlc1 binds to the cell division site. Mlc1 is the essential myosin light chain for Myo1 and Myo2, a myosin-V motor that is involved in vesicle and organelle transport [43], [44]. Mlc1 interacts with the first of the two IQ motifs of Myo1 and with the IQ motifs of Myo2 [43], [44]. However, both interactions are dispensable for Mlc1 recruitment to the cell division site and the assembly of an actin ring [44]. In contrast, the interaction of Mlc2 with the second IQ motif of Myo1 is shown to be essential and sufficient for the recruitment of Mlc2 to the cell division site [44]. It is shown that Mlc1 localization depends on septins [44], but it is not known whether Mlc1 binds directly to the septin scaffold or is recruited by a so far unknown septin binding protein. A recent report shows that Myo1 and Bni1 contribute to Mlc1 recruitment to the cell division site before and during cytokinesis [45].

Another essential factor for AMR assembly is the GTPase Rho1. Rho1 is recruited to the cell division site in its GDP-bound form by its guanine-nucleotide-exchange factors (GEF) through a process that involves Plk1 (polo-like kinase 1) mediated phosphorylation of the Rho1 GEFs [46]. Once activated at the cell division site, GTP bound Rho1 binds and activates the formins Bni1 and Bnr1, which in turn promote actin ring formation through their ability to nucleate actin filaments [47], [48], [49]. Bnr1 and Bni1 are functionally redundant, but show differential localization patterns. Bnr1 localizes to the cell division site in a septin dependent manner from G1 phase to telophase, and Bni1 from telophase to the end of cytokinesis [50], [51]. The recruitment of Bnr1 to the septin scaffold depends on different targeting domains [52]. However, the direct binding partner of Bnr1 and Bni1 at the bud neck is not known. The switch between Bnr1 and Bni1 is regulated by the phosphatase Cdc14 [53]. Consequently, the temperature sensitive MEN mutant *cdc15-2*, which fails to activate Cdc14 also fails to remove Bnr1 from the bud neck and to efficiently recruit Bni1 under restrictive temperatures [53]. The localization pattern and genetic evidences indicate that Bni1 is the major formin that contributes to actin ring formation [54]. Beside formins, tropomyosins and profilin contribute to actin cable formation and stabilization, and consequently, also to actin ring formation [47], [48], [55], [56]. The recruitment of tropomyosin Tpm2 to the cell division site depends on Rho1 activation [46].

Taken together, the assembly of the AMR is a complex process, which occurs stepwise throughout the cell cycle, but can be simplified to three key stages: (1) actin cable formation and stabilization at the cell division site by Rho1 activated formins, profilin and tropomyosin; (2) Iqg1 interaction with actin cables and contribution to actin ring formation and stabilization and (3) myosin light and heavy chains forming a template for Iqg1 mediated actin-ring formation.

## 3 AMR constriction and disassembly

It was originally believed that the AMR works like muscle-sarcomeres through a “filament-sliding” mechanism, in which myosin-II motors walk along anti-parallel organized actin filaments thereby generating the force that drives AMR constriction [57]. However, there are two major differences between the muscle-sarcomeres and the contractile AMR. First, the actin filaments in the AMR of mammalian and fission yeast cells appear to be organized in an isotropic rather than parallel or antiparallel manner [58], [59], [60]. How the actin cables are organized in budding yeast is unknown and comparative studies have not been performed to date. Second, the AMR disassembles during contraction, which is not the case for actomyosin filaments in sarcomeres [11], [13], [61], [62], [63]. These observations indicate that the AMR uses different mechanisms to drive constriction. A theoretical model suggests that filament dynamics in actin bundles can generate contractile stress [64]. In this model the authors propose that actin disassembly can contribute to force generation in the presence of end-tracking actin cross-linkers. Recent studies in budding yeast focused on this problem, investigating the contribution of the myosin motor domain and AMR disassembly to force generation and AMR constriction [10], [11], [12], [13], [44]. These studies demonstrate that the motor domain of myosin-II is not essential for cytokinesis [10], [12], [13]. However, the AMR constriction rate was slowed down by 20–40% [10], [11], [12], thus indicating that the motor domain contributes significantly to the process of ring constriction. The deletion of the regulatory light chain Mlc2 has a similar effect on AMR constriction [11], [44]. However, disrupting the interaction between Mlc2 and the IQ2 motif of Myo1 does not phenocopy the defect of Mlc2 deletion [44]. How Mlc2 contributes to AMR constriction is not currently understood. It is possible that the IQ2 motif has an auto-inhibitory function in the absence of Mlc2 [44]. The role of actin disassembly in AMR constriction was investigated by using the drug jasplakinolide, which stabilize actin filaments, and a mutant of cofilin (cof1-22), which is the main actin-depolymerization factor in yeast [11]. Both approaches show that actin depolymerization is important for normal AMR constriction rates. Mendes Pinto et al. show that deletion of the myosin-II motor domain and inhibition of actin depolymerisation have a syngeristic effect on AMR constriction. This result indicates that AMR constriction is driven by two independent processes that are mediated by actin depolymerisation and the motor domain of myosin-II. The defect of AMR constriction by blocking actin disassembly argues for a role of actin filament disassembly in driving AMR constriction, but can also be explained by different mechanisms. For example, AMR constriction is coupled to primary septum formation (see also following sections for mechanisms regulating primary septum formation). Thus, blocking or inhibiting primary septum formation could impair AMR constriction. Primary septum formation depends on vesicle trafficking along actin cables and exo- and endocytotic events. These processes depend on actin dynamics, which are severely impaired in cofilin mutants [65]. Therefore, inhibition of actin disassembly could rather reflect a defect in primary septum formation than in AMR constriction. If primary septum formation is a driving force for AMR constriction, then a defect in AMR disassembly could also be explained in a way that the non-contractile AMR inhibits or perturbs primary septum formation.

AMR constriction was shown to be under the control of the SCF(Grr1) ubiquitin ligase by promoting Hof1 degradation [66]. Another ubiquitin ligase, the anaphase promoting complex/cyclosome (APC/C) was reported to promote AMR disassembly by ubiquitination of Iqg1 and degradation by the proteasome [42]. Furthermore, it was shown that deletion of Cdh1, which is an activator of APC/C, prevents complete disassembly of Myo1, Mlc1, Mlc2 and Iqg1 at the cell division site [67]. However, this process does not impact AMR constriction [67]. Instead, it seems to be important to remove remnants of the constricted AMR from the cell division site to allow complete closure of the plasma membrane [67].

AMR constriction is a complex process and we are just beginning to understand the molecular mechanisms involved. The motor domain of Myo1, the regulatory myosin light chain Mlc2 and actin dynamics clearly contribute to AMR constriction. The molecular mechanism of the latter two and which role the primary septum plays remains to be fully elucidated.

## 4 AMR driven primary septum formation

### 4.1 Formation of the primary septum

**Fig. 3:**
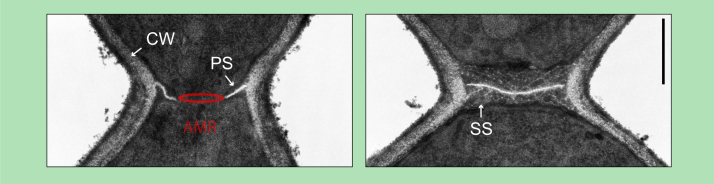
Electron micrograph of the bud neck region of Saccharomyces cerevisiae during primary septum (PS) formation (left panel) and after secondary septum (SS) deposition (right panel). The actomyosin ring (AMR), which is not visible in the shown electron micrograph, is illustrated as red ring. CW, cell wall. Scale bar: 0.5 μm.

AMR constriction is accompanied by centripetal formation of the primary septum (Fig. 2, Fig. 3). The primary septum consists of the polymer chitin (β-1,4 linked *N*-actylglucosamine). Chitin synthases are integral membrane proteins with multiple trans-membrane domains. Budding yeast has three chitin synthases (Chs1–Chs3). Chs2 is the major enzyme responsible for primary septum formation, whereas Chs1 and Chs3 function in cell wall repair and bud scar formation [68], [69], [70]. Once translated, Chs2 is stored in the endoplasmic reticulum. Phosphorylation of the N-terminus of Chs2p by the cyclin-dependent kinase Cdk1 prevents packaging into COPII vesicles and transport to the cell division site [71], [72], [73]. Upon mitotic exit, dephosphorylation of Chs2p by the phosphatase Cdc14p stimulates selection into vesicles and consequently its transport *via* the secretory pathway to the cell division site [73], [74], [75], [76]. The MEN kinases Cdc15 and Dbf2 regulate Chs2 recruitment to the cell division site [71], [77]. These observations can be partially explained by the function of Cdc15 and Dbf2 in activating the phosphatase Cdc14 [71], [77]. Recently, it was shown that the bud neck associated MEN kinase Dbf2 can directly regulate Chs2 dynamics, which supports its removal from the division site by the endocytic machinery [78]. Activation of Chs2 also depends on C2-domain protein Inn1 and is further supported by transglutaminase-like protein Cyk3 in an unknown manner [79], [80]. Taken together, primary septum formation is tightly linked to the cell cycle progression and involves temporal targeted secretion of Chs2 to the cell division site, where it is specifically activated by different mechanisms.

### 4.2 Interdependency of AMR constriction and primary septum formation

AMR constriction goes hand in hand with primary septum formation. This raises the question as to whether both processes are interdependent and whether a defect in primary septum formation can impair AMR constriction and *vice versa*. This would have a strong impact on the interpretation of data surrounding AMR constriction in different mutants. Hence, mutations that affect primary septum formation could be wrongly interpreted as mutations that directly affect the contractility and force generation of the AMR. These circumstances make it difficult to interpret data about proteins whose functions in cytokinesis are not completely understood. Among these proteins are the F-BAR protein Hof1, the C2 domain containing protein Inn1 and the transglutaminase-like protein Cyk3. These proteins are known to interact with each other [80], [81] and are thought to localize between AMR and plasma membrane during cytokinesis. Inn1 is targeted to the cell division site through four PXXP motifs, which interact with the SH3 domains of Hof1 and Cyk3. The interaction between Inn1 and Cyk3 is regulated by Cdk1-dependent phosphorylation and Cdc14-dependent dephosphorylation events [82]. The SH3 domain of Hof1 can also interact with Cyk3 [83]. Several lines of evidence support the idea that Inn1 and Cyk3 stimulate primary septum formation. First, the C2 domain of Inn1 is essential and sufficient for primary septum formation [80]. Second, a genetic suppressor screen identifies a hyperactive mutant of Chs2, which can rescue a lethal mutation in the C2 domain of Inn1 [79]. The same study shows that Inn1 interacts with Chs2 and can promote Chs2 activity *in vitro*. The role of Cyk3 is less clear, as it is not essential for primary septum formation [3], [84], however, overexpression of Cyk3 can rescue primary septum formation in the absence of Inn1 [80]. A role for Cyk3 in primary septum formation is also supported by the finding that hyperactive Chs2 genetically rescues defects associated with a Cyk3 deletion [79]. Whether Cyk3 and Inn1 have a direct function in regulating AMR contractility is not known. Hof1 was shown to bind to the AMR through a ring localization sequence (RLS) [85]. The direct interaction partner of Hof1 at the AMR was suggested to be Iqg1 [36]. Deletion of Hof1 causes severe defects in primary septum formation [85], though its precise role in primary septum formation is not fully understood. One possibility is that Hof1 promotes primary septum formation *via* the SH3 mediated interaction with Inn1 and Cyk3 [80], [83], [85]. However, the deletion of the SH3 domain of Hof1 causes no major defect in primary septum formation [85]. Phosphorylated Hof1 promotes AMR constriction [85]. However, it is not clear whether phosphorylated Hof1 directly activates AMR constriction or acts through primary septum formation in a similar manner as Cyk3 does. Interestingly, deletion of the primary septum synthesizing Chs2 and Inn1 seem to destabilize the AMR [75], [80], whereas deletion of Cyk3 significantly slows down AMR constriction, but does not destabilize the AMR [86]. The combination of Cyk3 depletion and a phospho-deficient Hof1 mutant prevents AMR constriction completely, but does not result in the destabilization of the AMR [86]. It is unclear why the AMR breaks in Inn1 or Chs2 deficient mutants that cannot form a primary septum, though one possibility is that the contractile forces tear the AMR apart when plasma membrane ingression is blocked, possibly due to defects in primary septum formation. Alternatively, Inn1 and Chs2 exert an AMR-stabilizing function.

Cells without Myo1 can form a primary septum [12], though Myo1, Iqg1 and consequently actin cables are important for guiding primary septum formation during cytokinesis [12]. Mutants lacking myosin form a primary septum, which often grows toward the cell body rather than in a centripetal manner. The tail of Myo1 forms a scaffold for Hof1 and Inn1 and is sufficient to guide centripetal primary septum formation [12], [13]. Interestingly, deletion of Myo1 or just the motor domain results in formation of more than one primary septum [12], though how this translates into the formation of a single primary septum is not known.

Taken together, efficient AMR constriction and primary septum formation are interdependent. It appears that the primary function of the AMR is to guide centripetal septum formation. On the other hand septum formation is important to stabilize AMR constriction during cytokinesis.

### 4.3 The molecular linker between AMR and plasma membrane

To be able to guide primary septum formation, the AMR must be linked to the plasma membrane. Two ways of interaction could be imagined. The AMR could be either linked to the lipid bilayer, or to an integral or peripheral membrane binding protein. Inn1 was originally described to couple AMR constriction with furrow ingression *via* its plasma membrane interacting C2 domain and an AMR interacting tail [87]. C2 domains are known to interact with lipids, however, the C2 domain of Inn1 shows no lipid binding activity [80], but interacts and activates the primary septum forming enzyme Chs2, which is an integral membrane binding protein [79]. The proline-rich tail of Inn1 interacts with the SH3 domains of Hof1 and Cyk3 [80], [81]. Finally, Hof1 binds to the AMR *via* a ring localization sequence (RLS) during AMR contraction. This domain was shown to form together with Iqg1 and Mlc1 a trimeric complex, which is part of the AMR [36]. Thus, Hof1 might form a bridge between AMR (Hof1-Iqg1-Mlc1 complex) and the plasma membrane *via* the septum promoting proteins (Hof1-Inn1-Cyk3) and Chs2 (Fig. 2). Interestingly, Hof1 can also directly interact with the plasma membrane through its N-terminal F-BAR domain [88]. A recent study shows that the transmembrane protein Sho1 interacts *via* its SH3 domain with Hof1, Cyk3 and Inn1 [83]. Thus, at least three membrane binding proteins (Chs2, Hof1 and Sho1) might contribute to connect the AMR with the plasma membrane during cytokinesis. Furthermore, it is suggested that the SH3 domains of Sho1, Hof1 and Cyk3 form a complex network of interactions at the cell division site [89]. This might explain why the perturbation of single SH3 mediated interactions rarely causes any major defects in cytokinesis [80], [83], [85]. Thus, the complex network of interactions between the plasma membrane and the AMR could form redundancy to provide a certain amount of robustness to the system.

## 5 Secondary septum formation and abscission

After AMR contraction, the secondary septum is deposited on both sides of the primary septum (Fig. 3). The secondary septum is similar to the cell wall and consists mainly of β-1,3-glucan and mannoproteins [90]. The major glucan synthase involved in secondary septum formation is Fks1 [91]. Fks1 localizes at the cell division site [92] and is under the control of the GTPase Rho1 [93]. Rho1 also recruits Chs3, which contributes to secondary septum formation, to the cell division site [69], [94], [95]. Rho1 is further essential for exocytosis [96] and formin mediated actin cable formation for trafficking and to form the AMR [47], [97]. Thus, Rho1 is a major regulator of cell division. Rho1 activity is controlled by the guanine exchange factors (GEF) Tus1 and Rom2 and by the GTPase activating protein (GAP) Lrg1. Tus1 recruits Rho1 to the cell division site, which is important for AMR assembly and contraction [46]. Rom2 localizes at the cell division site after AMR contraction and might be involved in secondary septum formation [98]. Maintenance of Rho1 at the cell division site after AMR contraction depends on its C-terminal membrane binding domain and on phosphatidylinositol 4,5-bisposphate [94]. Rho1 activation and its function in secondary septum formation is further under the control of Rho1 binding protein Gps1 [99], [100]. During AMR contraction, Rho1 is inhibited by Cyk3 to prevent premature secondary septum formation [84]. Timely recruitment of Cyk3 to the cell division site is controlled by the MEN [77]. After AMR contraction, Rho1 is negatively regulated by the GTPase activating protein (GAP) Lrg1, which is important to prevent massive deposition of cell wall material resulting in abscission defects [101], [102]. The second major Rho GTPase Cdc42 is inhibited at the cell division site during and immediately after cytokinesis to prevent diverse malfunctions [84], [99], [100], [103]. Finally, the daughter cell is separated from the mother cell through partial degradation of the primary and secondary septum by the chitinase Cts1 and several glucanases [104]. The expression of these hydrolases and of the Rho1 promoting factor Gps1 is under the control of the transcription factors Ace2 and Swi5, which are activated during mitotic exit [105], [106], [107]. Whether Rho1 directly regulates cell cleavage is not known. However, Gps1 deletion results in the formation of multi-budded cells that fail to cleave indicating that Rho1 is also involved in cell cleavage [99].

Taken together, Rho1 is in addition to its function in AMR assembly the major regulator of secondary septum formation. This requires sequential Rho1 activation and inhibition steps in a spatio-temporal manner to allow the successive formation of the AMR and secondary septum and subsequent cell separation.

## 6 Conclusions and perspectives

Research over the last two decades has identified essential components of the cell division machinery as well as their complex interactions throughout the cell cycle. Structural information, investigations into the molecular mechanisms and of key and regulatory components have given insight into how the AMR drives cytokinesis. However, we do not completely understand the basic mechanisms that drive and coordinate AMR constriction and septum formation. A future milestone in the field will be to establish an *in vitro* system that is able to simulate *in vivo* characteristics of the cell division machinery. This would allow one to investigate the underlying mechanisms in a fully tunable manner. *In vivo* evidence has demonstrated that the formation of extracellular matrix and the involved membrane associated proteins might be essential for the function of the AMR and even for its assembly. The implementation of these aspects in an *in vitro* system will be challenging. Nevertheless, recent progresses in *in vitro* systems have shown that many of the challenges can be addressed with a new array of tools [108], [109], [110], [111], [112].

## Figures and Tables

**Table 1:** Proteins involved in cytokinesis.

Generic name	Standard name	Functions	References
Actomyosin-ring components			
Actin	Act1	ATPase; structural component involved in actin ring assembly; filament formation	[38], [39], [113]
Myosin-II heavy chain	Myo1	ATPase motor activity; involved in AMR assembly via tail mediated scaffolding	[39], [113]
Essential myosin light chain	Mlc1	Recruitment of Myo1 and Iqg1; stabilization of AMR during constriction	[35], [36], [37], [43], [44], [114] [12]
Regulatory myosin light chain	Mlc2	Binds to myosin-II heavy chain, involved in AMR constriction	[44]

Actomyosin-ring assembly			
Septins	Cdc3, Cdc10, Cdc11, Cdc12 Shs1	GTP binding proteins, filament formation; scaffold for AMR assembly	[16], [18], [115], [116]
Septin-interacting protein	Bni5	Myo1 recruitment before cytokinesis; crosslinks septin filaments	[12], [30], [31]
Formins	Bni1, Bnr1	Nucleator of actin filaments	[47], [50], [54]
Rho GTPase	Rho1	Formin activation; actin ring assembly	[47]
Guanine nucleotide exchange factor	Tus1, Rom1, Rom2	Rho1 activation; involved in actin ring assembly	[46]
Tropomyosins	Tpm1, Tpm2	Binds and stabilizes actin filaments	[56]
Profilin	Pfy1	Actin binding protein essential for actin filament nucleation	[48]
IQGAP	Iqg1	Actin filament organization; interacts with Mlc1; AMR stability during constriction	[12], [34], [35], [36], [37], [38]

Actomyosin-ring disassembly			
Cofilin	Cof1	Binds both actin monomers and filaments, actin filament severing and depolymerization	[11]
Anaphase promoting complex	APC(Cdh1)	Ubiquitin ligase that promotes the proteasome mediated degradation of AMR remnants after constriction	[42], [67]
SCF ubiquitin ligase	SCF(Grr1)	Ubiquitin ligase that promotes Hof1 degradation; contributes to AMR constriction	[66]

Primary septum formation			
Chitin synthase 2	Chs2	Synthesis of the primary septum	[68], [69], [70], [74]
C2-domain protein	Inn1	Chs2 activation; interacts with Hof1 and Cyk3	[77], [79], [80], [81], [82], [87]
Transgluaminase-like protein	Cyk3	Chs2 activation; Rho1 inhibition; interacts with Hof1 and Inn1	[79], [80], [81], [84], [117]
F-BAR protein	Hof1	Primary septum formation, AMR constriction; interacts with Inn1, Cyk3, septins and Sho1	[36], [54], [80], [85], [118] [83]

Secondary septum formation			
Rho GTPase	Rho1	GTPase that regulates secondary septum biogenesis by Fks1 activation	[93]
1,3-β glucan synthase	Fks1, Fks2	catalytic subunit of 1,3 beta-D-glucan synthase	[91], [119]
GTPase-mediated polarity switch 1	Gps1 (Aim44)	Rho1 recruitment to the cell division site	[99], [100]
Chitin synthase 3	Chs3	Chitin synthesis	[69], [74], [94]

Cell separation			
Chitinase	Cts1	Plays a major role in cell separation after cytokinesis by chitin hydrolysis	[120], [121]
Glucanases	Eng1, Egt2	Plays a major role in cell separation after cytokinesis by glucan hydrolysis	[122], [123]

**Table 2:** Kinases and phosphatases regulating cytokinesis.

Generic name	Standard name	Function	References
Kinases and regulatory subunits			
Polo kinase	Cdc5	Hof1 regulation, Rho1 activation	[46], [85]
NDR/LATS related kinase complex	Dbf2-Mob1	Hof1 regulation, Chs2 regulation	[78], [85], [86]
NDR/LATS related kinase complex	Cbk1-Mob2	Regulates cell separation via the transcription factor Ace2	[124], [125]
Mitotic cyclin dependent kinase	Cdk1-Clb2	Chs2 trafficking, Iqg1 stability, Hof1 regulation, Inn1-Cyk3 interaction	[40], [73], [76], [82], [85]

Phosphatases and regulatory subunits			
Cdc14 family	Cdc14	Chs2 trafficking, Iqg1 stability, Inn1 localization, Inn1-Cyk3 interaction	[41], [76], [82], [126], [127]
PP2A family	Rts1	Septin dynamics	[128]
